# Observation of the Multilayer Growth Mode in Ternary InGaAs Nanowires

**DOI:** 10.1021/acsnanoscienceau.2c00028

**Published:** 2022-08-30

**Authors:** Robin Sjökvist, Marcus Tornberg, Mikelis Marnauza, Daniel Jacobsson, Kimberly A. Dick

**Affiliations:** † Centre for Analysis and Synthesis, Lund University, Box 124, 22100 Lund, Sweden; ‡ NanoLund, Lund University, Box 118, 22100 Lund, Sweden; § National Centre for High Resolution Electron Microscopy, Lund University, Box 124, 22100 Lund, Sweden

**Keywords:** *In situ* TEM, Nanowire, MOCVD, Crystal growth dynamics, InGaAs, III−V, Ternary semiconductor

## Abstract

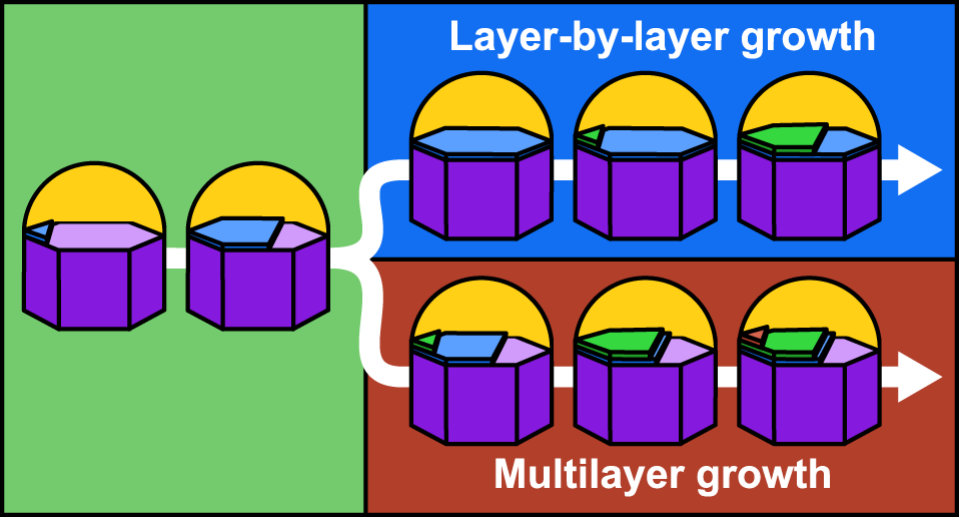

Au-seeded semiconductor nanowires have classically been considered to only grow in a layer-by-layer growth mode, where individual layers nucleate and grow one at a time with an incubation step in between. Recent *in situ* investigations have shown that there are circumstances where binary semiconductor nanowires grow in a multilayer fashion, creating a stack of incomplete layers at the interface between a nanoparticle and a nanowire. In the current investigation, the growth behavior in ternary InGaAs nanowires has been analyzed *in situ*, using environmental transmission electron microscopy. The investigation has revealed that multilayer growth also occurs for ternary nanowires and appears to be more common than in the binary case. In addition, the size of the multilayer stacks observed is much larger than what has been reported previously. The investigation details the implications of multilayers for the overall growth of the nanowires, as well as the surrounding conditions under which it has manifested. We show that multilayer growth is highly dynamic, where the stack of layers regularly changes size by transporting material between the growing layers. Another observation is that multilayer growth can be initiated in conjunction with the formation of crystallographic defects and compositional changes. In addition, the role that multilayers can have in behaviors such as growth failure and kinking, sometimes observed when creating heterostructures between GaAs and InAs *ex situ*, is discussed. The prevalence of multilayer growth in this ternary material system implies that, in order to fully understand and accurately predict the growth of nanowires of complex composition and structure, multilayer growth has to be considered.

## Introduction

Atomic scale control is a necessity to construct effective and precise devices operating at the nanoscale. In the case of III–V semiconductor nanowire growth there are many possibilities for such delicate device design, where the composition, crystal structure, and interfacial sharpness of the nanowire segments ultimately determine the performance.^1,2^ A key factor for the control and design of nanowires is our understanding of the vapor–liquid–solid (VLS) growth process, the most commonly used method for nanowire growth. In VLS growth, a liquid nanoparticle collects material from the surrounding vapor, which enables the nucleation and growth of III–V material at the interface between the nanoparticle and the solid nanowire. The current understanding is that the growth proceeds one III–V bilayer at a time. During the growth of a layer, further nucleation is suppressed since the chemical potential difference, or supersaturation, of the elements in the nanoparticle with respect to the solid nanowire is decreased. After the layer finishes, there is a possibility for the nanoparticle to build up the supersaturation again by collecting more growth material, before the nucleation and growth of the next layer.^3,4^ The process of a waiting period (incubation) followed by solidification of material (layer propagation) results in a cyclic layer-by-layer growth process. However, recent *in situ* investigations have shown the presence of multilayer growth, where multiple layers nucleate and grow simultaneously.^5,6^

So far, the presence of multilayers has been shown for a few binary compound nanowires.^5,6^ Binary nanowires consist of a 1:1 combination of an element from group III and one from group V of the periodic table, forming compounds such as GaP and InAs. However, one should also consider the presence of multilayers, and their implications for the properties, in the technologically important ternary compound nanowires. In ternary nanowires, another element of either group III or V is incorporated, and the final properties are weighted combinations between the two resulting binaries (e.g., In_*x*_Ga_1–*x*_As or GaAs_*x*_Sb_1–*x*_). Since compositional control is central for the ability to tailor ternary nanowires to specific applications,^7,8^ ternary compositions have been investigated both *ex situ*(9) and *in situ*.^10^ It has recently been shown that the layer evolution in ternary nanowires can determine and affect the resulting composition,^11^ which gives a reason to believe that the presence of multiple growing layers will affect the overall composition in the segment.

Another way to tailor the properties of nanowires is to incorporate different segments into the structure, forming heterostructures where the ternary composition or III–V material changes abruptly along the nanowire. The goal of research on heterostructures in nanowires is to be able to precisely control the length and sharpness of each segment present, in order to create complex structures such as embedded quantum dots or 3D structures.^2,12,13^ To achieve this type of growth, control of the individual layers in the structure is crucial. For some combinations, e.g. the GaAs to InAs transition, however, the lattice mismatch between the two materials is significant, making the formation of sharp switches difficult and often resulting in a change in growth direction (kinking),^14^ a ternary transition region,^15^ or growth along one of the side facets of the nanowire.^16,17^

In this study, environmental transmission electron microscopy (TEM) has been used to investigate the growth dynamics of InGaAs nanowires *in situ*. This was done in order to study the growth processes occurring in ternary transition regions between GaAs and InAs. The study revealed the occurrence of multilayer growth at intermediate ternary compositions, where stacks of multilayers much larger than those previously reported are observed.^6^ We show that multilayers form in conjunction with crystal defect formation, sharp and diffuse compositional changes, and kinking behavior. Even small changes in composition seem to facilitate multilayer growth and can thus give clues about the difficulty in achieving a sharp GaAs to InAs heterostructure. We also observe redistribution of material between the layers of a multilayer stack, indicating that the composition of the layers can change even after initial solidification. Further study of multilayers will therefore be important to achieve compositional and structural control of InGaAs nanowires.

## Results

**Figure 1:**
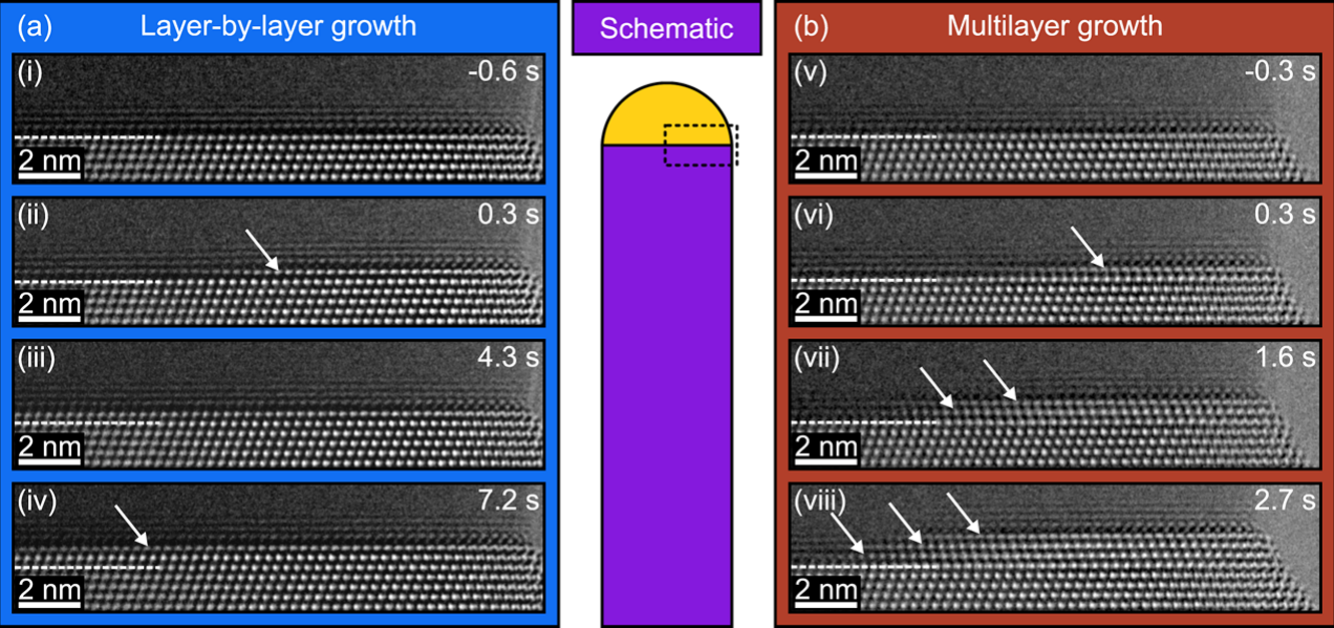
*In situ* time series of typical layer-by-layer growth and multilayer growth in nanowires. The dashed box in the schematic indicates the region of the nanowire from where the micrographs in (a) and (b) are recorded. (a) shows layer-by-layer growth, where the time stamp in micrographs (i–iv) tells the time relative to the nucleation of the layer that is shown growing in (ii). The frames show the interface before nucleation (i), after nucleation (ii), during incubation (iii), and after the next nucleation (iv). Similarly, (b) shows the multilayer growth process, where the times tamp in micrographs (v–viii) again is relative to the nucleation of the first layer: (v) is before nucleation, (vi) is after the nucleation of the first layer, (vii) is after the nucleation of the second layer, and (viii) is after the nucleation of the third layer. The white dashed lines in all micrographs are guides to see the addition of layers during the growth, while the white arrows point to the currently growing layers. All micrographs are constructed by averaging 5 frames from a 10 frames per second (fps) video, with the time stamp representing the central frame. The contrast observed over the topmost layer in the micrographs (and most subsequent micrographs in this publication) is attributed to the presence of liquid ordering within the Au nanoparticle.^18^

In order to discuss multilayers, a demonstration of what they are is necessary. Figure 1 shows several frames extracted from videos recorded using TEM (details can be found in the Experimental Section, as well as in section SI 1 in the Supporting Information). The frames depict the liquid–solid interface of a nanowire grown *in situ*, where Figure 1a shows the nanowire growing in the traditional layer-by-layer growth mode and Figure 1b shows it growing through multiple nucleation events, resulting in multilayer growth. The growth in Figure 1a therefore follows the incubation and layer propagation process, where the liquid nanoparticle, residing on top of the nanowire, increases its supersaturation during the incubation step, leading to a nucleation event followed by layer propagation. Figure 1a-i illustrates the interface during one such incubation period, 0.6 s before the nucleation of the layer seen in Figure 1a-ii (indicated by the time stamp). After the growth of the layer shown in panel Figure 1a-ii there is again a significant incubation time (Figure 1a-iii) before the nucleation and growth of the next layer, shown in Figure 1a-iv.

The layer propagation and incubation process detailed in Figure 1a is in contrast with the multilayer growth behavior shown in Figure 1b. The multilayer growth process involves several different layers (indicated by arrows) growing simultaneously, and therefore new nucleation events occur before the previous layer has grown to completion. The multilayer growth starts out with a flat interface shown in panel Figure 1b-v, similarly to Figure 1a-i in the layer-by-layer case. Figure 1b-vi shows a growing layer 0.3 s after nucleation, while Figure 1b-vii shows the same layer, still unfinished, along with a new layer nucleated on top. In Figure 1b-viii another layer is seen growing, giving a total of three simultaneously growing layers, forming a staircaselike structure of atomic steps at the interface. As will be discussed later, the growth front of the multilayer stack is highly dynamic, meaning that the angle the atomic steps form to the rest of the liquid–solid interface changes frequently during the growth of the multilayer segment.

An important distinction between the multilayer and layer-by-layer cases is that for multilayer growth there is no separate incubation period between layers. This means that enough material must be collected during the layer propagation to facilitate the nucleation of the next layer. While growth material is assumed to be collected during the layer propagation step in regular layer-by-layer growth as well, the supersaturation is not high enough to initiate another nucleation until after the completion of a layer.

**Figure 2:**
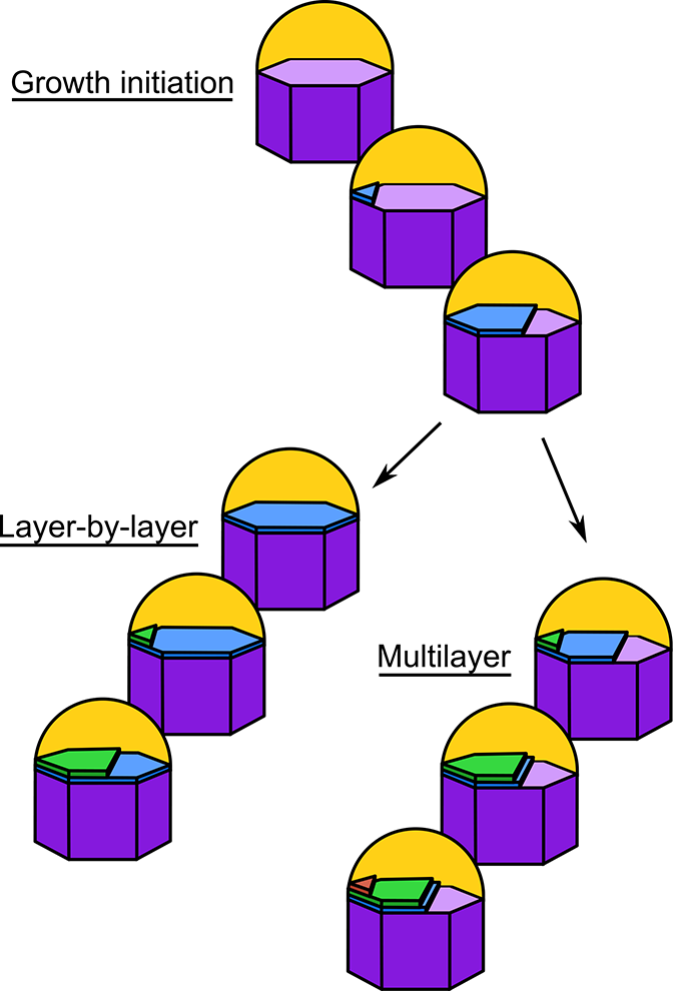
Schematic detailing the layer progression in both the layer-by-layer growth mode and the multilayer growth mode, highlighting their differences and similar origin. Note that the shape evolution of the individual layers is not treated in this study, and as such the schematics are showing a simplification.

From Figure 1 it becomes evident that the multilayer and layer-by-layer growth modes start out the same way, with the nucleation of a single layer at the interface. This initial common origin is illustrated in the growth initiation part of Figure 2, which then proceeds to split up into the two growth modes. The growing layer has the possibility of either spreading to completely cover the interface and follow the layer-by-layer path or nucleating a new layer and follow the multilayer path.

### Dynamics of Multilayer Stacks

**Figure 3:**
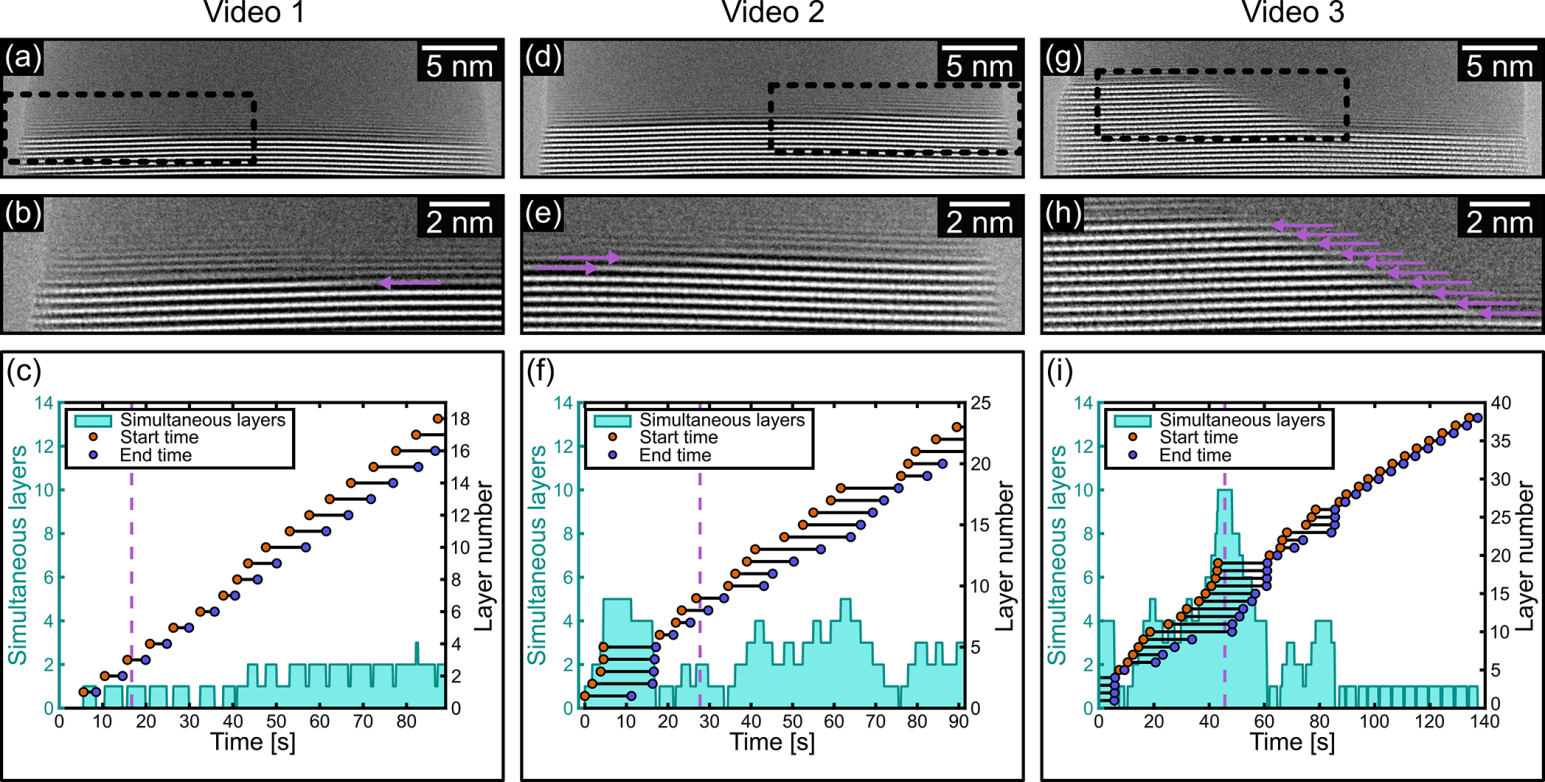
Temporal evolution of a nanowire segment that includes significant multilayer growth. Each column represents one of three videos recorded during the growth of the segment, indicated by the labels. (a, d, g) show frames extracted from the videos, where different amounts of layers are currently growing. The times of the frames are indicated by the purple dashed lines in (c, f, i), respectively. The dashed regions in (a, d, g) indicate the part of the image that is represented in (b, e, h), respectively, in which growing layers are indicated by purple arrows. (c, f, i) show the start (orange circles) and end (blue circles) times of the layers recorded relative to the start of each video, as well as the current number of simultaneously growing layers (cyan bars). Sections of the original videos can be found in Videos 1–3 in the the Supporting Information, respectively.

To give insight into how the number of layers in a multilayer stack varies over time, a summary of an extensive multilayer growth event under stable surrounding conditions is presented in Figure 3. The figure is split into three columns, each representing one of three videos recorded in sequence from the growth of a nanowire segment where multilayer growth was prominent. A section of each video is supplied as Videos 1–3, respectively, in the Supporting Information. The leftmost column (Figure 3a–c) shows the behavior at the start of the multilayer segment. Figure 3a depicts a frame extracted from the video, showing a single growing layer. The dashed region of Figure 3a shows the area presented in Figure 3b, which gives a better view of the growing layer (indicated by the purple arrow). Figure 3c shows a summary of the layers encountered in the video, with start and end times as well as a cyan bar graph that shows the number of simultaneously growing layers. Here it can be seen that initially the nanowire grows one layer at a time with incubation periods in between, following the established layer-by-layer growth mode. The purple dashed line shows the time of the frame shown in Figure 3a,b, indicating which layer is shown growing (layer 3) and the number of layers growing simultaneously (1 in this case). At layer 8 (around the 40 s mark in Figure 3c), we see the first occurrence of multilayer growth, where the start of the next layer comes before the end of the previous layer. Hence, the cyan bar graph shows that we have two layers growing simultaneously for a short period of time. This behavior continues for the rest of the video, with a very short segment showing three simultaneously growing layers after the 80 s mark. As we enter the multilayer growth regime, it is important to note that the time between the start (orange circles) and end (blue circles) of each layer increases. This can be attributed to a competition for material as multiple layers grow simultaneously, since the supply of growth material is kept constant.

The middle column of Figure 3 (Figure 3d–f) shows data extracted from Video 2 in the series, which starts a few seconds after the end of Video 1. Here, we see a big difference in the behavior of the growth. In this segment, there is almost always more than one layer growing at the same time, and the number varies greatly (indicated by the cyan bar graph in Figure 3f). As a result, the absolute time it takes for each layer to grow also varies. Figure 3d,e shows the smallest possible multilayer stack, with only two simultaneously growing layers (layers 8 and 9 indicated by the purple dashed line in Figure 3f).

In the rightmost column, data from Video 3, which starts a few seconds after the end of Video 2, are presented (Figure 3g–i). In this segment, the largest multilayer stack is observed, indicated by the purple dashed line in Figure 3f, and a frame is shown in Figure 3g,h. Here, 10 layers (layers 10–19) are growing simultaneously. The bottommost layer in this stack is the layer that takes the longest time to complete out of all other layers in the three videos presented. After this multilayer segment the nanowire experiences another region of multilayer growth around the 70 s mark before going back to normal layer-by-layer growth, exhibiting once again incubation between the nucleation and growth of singular layers.

X-ray energy dispersive spectroscopy (XEDS) was performed on the nanowire presented in Figure 3, before and after the multilayer segment, showing a change in composition from roughly In_0.5_Ga_0.5_As to In_0.65_Ga_0.35_As in the nanowire. XEDS measurements performed on the multilayer segment after its growth indicate that the average composition is intermediate to the compositions of the surrounding segments. In section SI 2 in the Supporting Information, a geometric phase analysis (GPA)^19^ treatment of a frame in each video is supplied, in order to show that the lattice parameter of the nanowire does not change drastically across the multilayer region. This indicates that the compositional change is gradual, which is to be expected for the growth conditions used. The nanoparticle composition was also measured before and after the multilayer growth, which revealed that the nanoparticle composition was more consistent, with Ga < 3 atom % and In > 40 atom %, similar to earlier *in situ* studies of In_*x*_Ga_1–*x*_As growth.^10^

**Figure 4:**
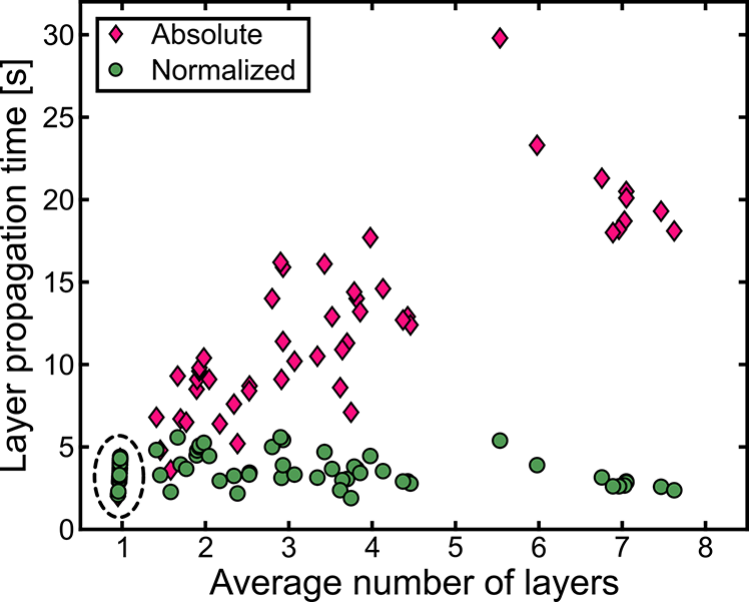
Layer propagation time as a function of the average number of layers that are growing simultaneously. The magenta diamonds show a summary of the absolute time it takes for each layer presented in Figure 3 to grow. In order to remove the contribution to the layer propagation time caused by the presence of multiple growing layers, the green circles were constructed (by normalizing to the average number of layers growing simultaneously). The two data sets overlap in the circled area, which represents the layer-by-layer growth mode. The green circles show that the average time it takes for a layer to grow in the multilayer growth mode is not very different from the time it would take in the layer-by-layer mode.

In order to learn more about the implications this multilayer growth has on the evolution of the nanowire, the layer propagation times of the individual layers were studied further. Figure 4 shows the layer propagation time of individual layers as a function of the average number of layers that grow simultaneously during the growth of said layer. The diamonds represent the measured layer propagation times of the layers. It becomes evident that the layer propagation time of a specific layer increases if more layers grow simultaneously. The question is whether or not this affects the overall growth rate of the nanowire. The circles in Figure 4 show the normalized propagation time and were constructed by dividing the values of the diamonds with the average number of layers growing simultaneously. This means that the growth time of singular layers in stacks of multilayers could be extracted. Focusing on the data present for layers growing in the layer-by-layer mode (average number of layers 1, indicated by the dashed circle), we see that there already is a spread in the layer growth time without multilayers being present. However, as the number of simultaneously growing layers increases, there is little change in either the spread or absolute values of layer propagation times. For the layers growing in the layer-by-layer mode, the incubation time is mostly small compared to the layer propagation time under the growth conditions used here (see for example the layers growing at times larger than 90 s in Figure 3i). This suggests that the overall growth rate is unchanged when the number of simultaneously growing layers is increased, which implies that multilayer growth is not a hindrance to growth but rather affects where the incoming material is incorporated. This also means that simply observing the length of the nanowire postgrowth would not reveal whether or not multilayer growth has occurred, showing the importance of studying this effect *in situ*.

### Mechanisms of Multilayer Propagation

**Figure 5:**
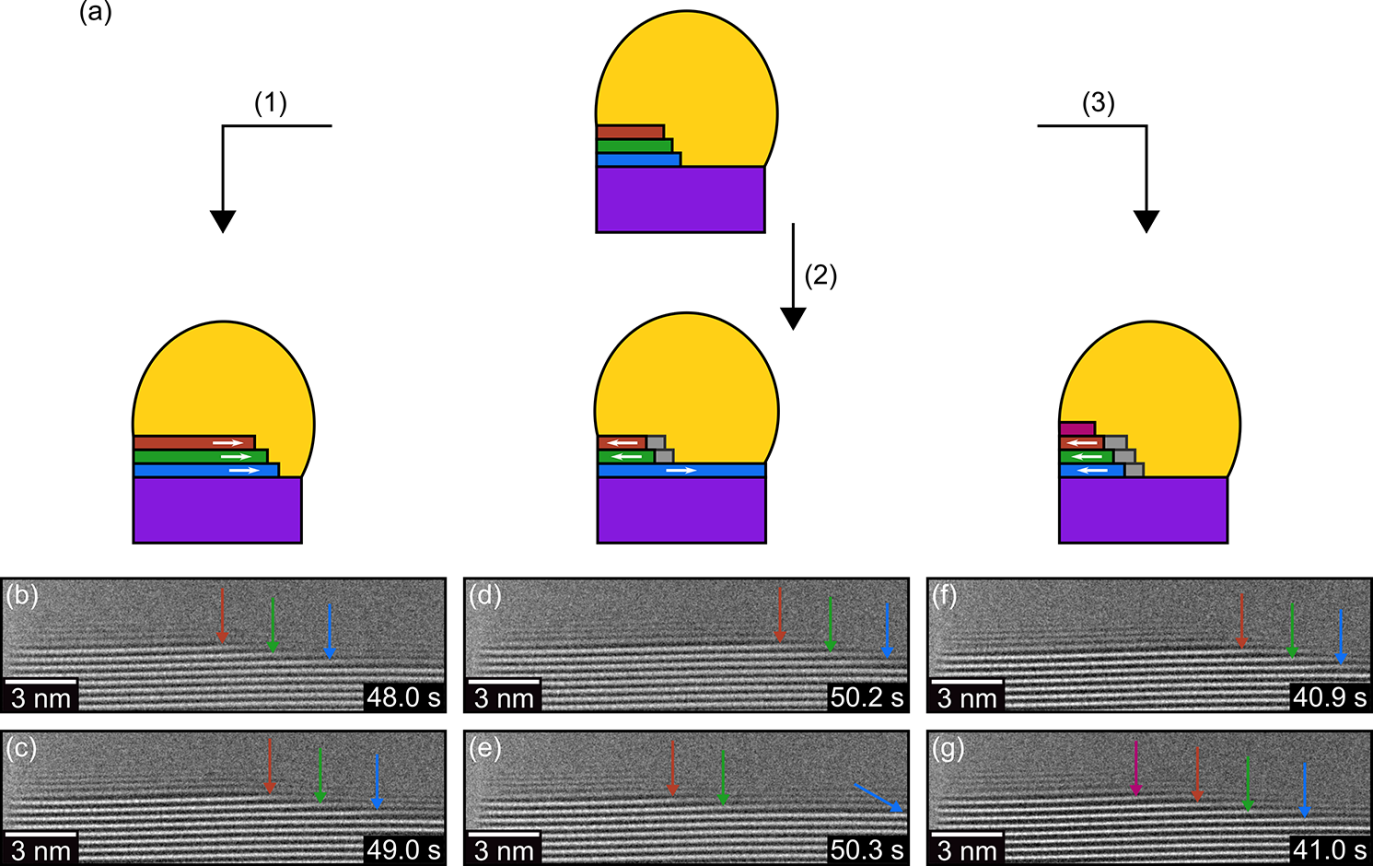
Three dynamic processes observed at the growth front of multilayer stacks. (a) shows a schematic of the three processes: (1) simultaneous growth of all layers, (2) the rapid completion of the bottommost layer, while parts of the other layers are consumed, and (3) the nucleation of a new layer in the stack, while parts of the other layers are consumed. (b) and (c) show micrographs of process 1, with color-coded arrows indicating the growth front of the respective layers. Similarly, (d) and (e) show micrographs detailing process 2 and (f) and (g) show micrographs detailing process 3.

The results from Figure 4 show that the overall layer propagation time is similar for layer-by-layer and multilayer growths. However, Figure 4 also shows that each individual layer in the multilayer stack exists as an incomplete layer for much longer than in the layer-by-layer case, meaning that there is a longer time during which attachment and detachment of atoms can occur to a particular layer. During layer propagation in the regular layer-by-layer growth of nanowires, the addition of atoms at the growth interface of the layer typically occurs at a steady rate.^20^ The highly dynamic behavior of the multilayer growth mode, however, was found to destabilize this process for the individual layers. In general, three different processes affecting the incorporation of atoms at the growth front were observed, presented in Figure 5.

Figure 5a shows schematics detailing the different processes found to occur, while Figure 5b–g show micrographs extracted from the same video as presented in Figure 3d–f. Process 1 in Figure 5a shows a situation where all the layers in the stack grow simultaneously. The same process is depicted in the micrographs shown in Figure 5b,c, which are 1 s apart (see the time stamp). The colored arrows indicate the growth front of the individual layers (propagating from left to right), and it is evident that the growth front of each layer is moved forward between Figure 5b and Figure 5c. The rate of layer propagation for individual layers is slow in this case, since the whole stack is growing simultaneously. However, all individual layers in the stack do not necessarily grow at the same rate during this process, meaning that there typically does not appear to be any extended periods where the growth front forms a preferred facet. This is seen more clearly in video format (Video 3).

Process 2 shown in Figure 5a shows a single-layer completion, where the bottommost layer in the stack rapidly finishes. Rapid completion of the bottom layer has been observed to occur even when the bottommost layer has only been approximately 50% complete prior to the initiation of process 2. As is indicated by the gray outlines in the schematic, this process often involves the consumption of parts of the previously initiated layers in the stack, meaning that solid material is moved between the layers. The movement of material can be observed in the micrographs presented in Figure 5d,e, where the upper layers (indicated by red and green arrows) retract between the frames and the bottom layer (blue arrow) moves out of frame as it proceeds to completion. The time difference between these two micrographs is only 0.1 s (see time stamps), which was the temporal resolution at which the video was recorded; thus, the actual speed of the process could be faster. This means that the temporal resolution does not allow us to discern the full process of the material movement; whether the initial material is first dissolved in the liquid or if the movement occurs via interlayer diffusion is unclear.

Process 3 in Figure 5a details the nucleation of a new layer to the stack, which, similarly to process 2, often involves the redistribution of material from the other layers in the stack. However, as is indicated by the differently colored arrows in Figure 5f,g, the consumption of already growing layers is not as drastic as for process 2. The time difference between the two frames is again 0.1 s, similar to earlier observations of nucleation events.^3^

Process 1 is the process with the least drastic changes to the structure of the growth interface, and it is the way the multilayer growth progresses between the occurrences of the more rapid processes detailed in 2 and 3. If process 1 is followed to completion and all layers finish, we end up in a situation where the interface between the nanowire and nanoparticle again is flat and an incubation step follows before growth is reinitiated. As was outlined in Figure 2, this could lead to the nanowire returning to the more typical layer-by-layer growth or initiate a new multilayer growth sequence.

The irregularities manifesting for multilayer growth are sure to have implications for the resulting structure and composition of the nanowire. The rapid completion of a layer shown in process 2 could for example allow the incorporation of defects within the layer, while the relocation of material in processes 2 and 3 allows the layer to change parts of its composition even after the initial solidification. There is also a possibility that the fast completion can facilitate the formation of stacking faults in the nanowire structure.

### Multilayer Growth at Heterostructures

As was previously mentioned, it has been reported for binary nanowires that multilayer growth can occur in conjunction with changes in the crystal structure.^5^ This is also true for ternary nanowires, as can be seen in Figure 1b, where the first layer in the multilayer stack nucleates as a twinned layer with regard to the already existing crystal. The following layers nucleate in the same twinned configuration as the first layer in the stack, creating a homogeneous segment. However, there are also examples of multilayer growth initiation without the presence of any kind of crystallographic perturbation, which implies that defects in the crystal structure are not a necessity for multilayer growth (see section SI 3 in the Supporting Information).

**Figure 6:**
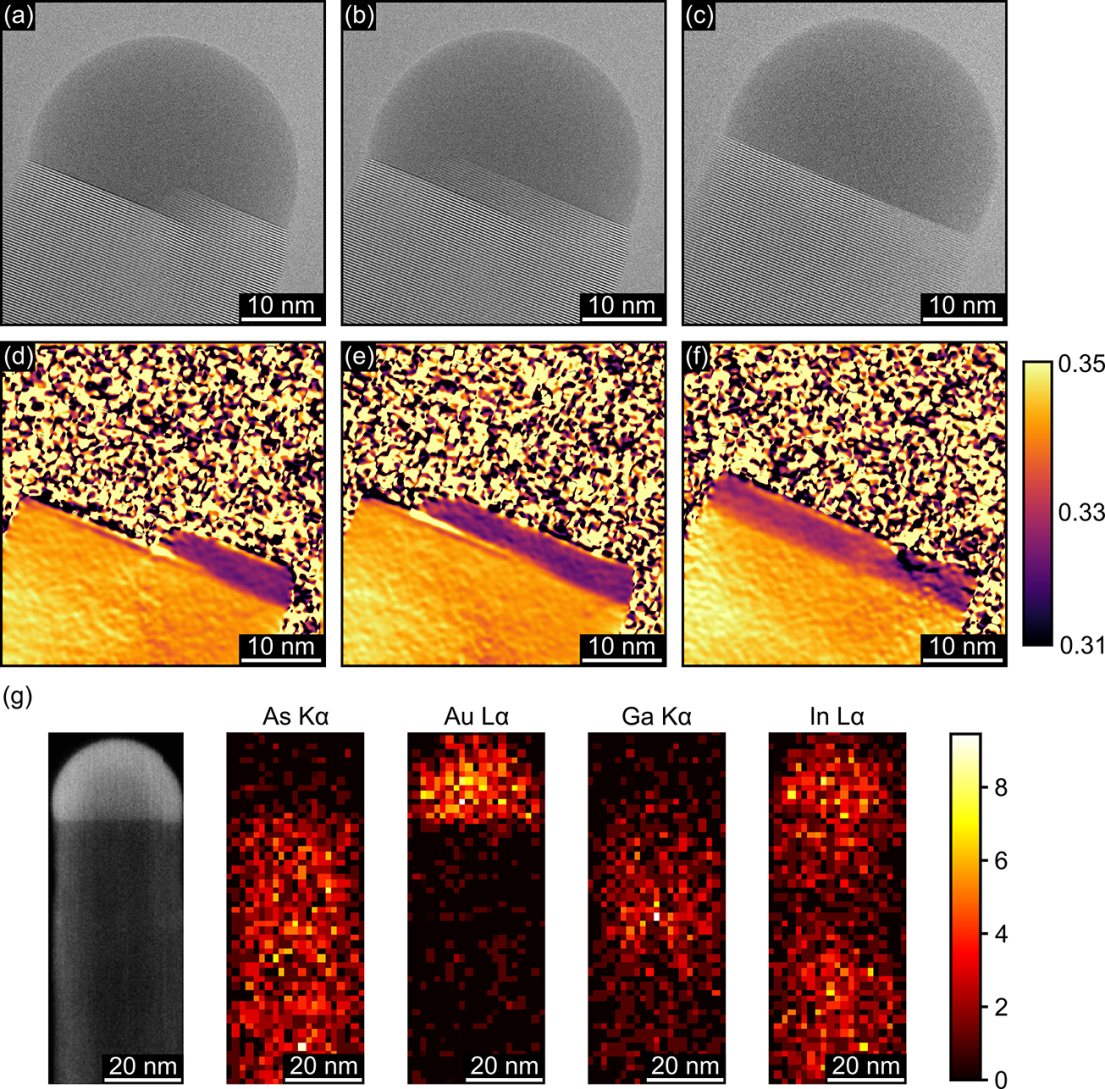
Multilayer event occurring in conjunction with a sharp compositional change along the growth direction of the nanowire. (a–c) show frames extracted from a video of a nanowire where a large multilayer stack is growing. In (c) the segment is completed. (d–f) show the same frames after GPA treatment, where the color is representing the interplanar distance in the growth direction in nanometers (see color bar). A smaller distance (purple) corresponds to more Ga rich InGaAs. (g) was constructed from a postgrowth XEDS analysis and shows qualitatively the locations of the different elements in the nanowire. The color bar indicates the intensity of the respective peaks in the different regions of the nanowire, and the region of high Ga intensity corresponds to the multilayer segment.

The results from Figure 3 show that the composition of the nanowire may change in conjunction with the multilayer growth event, which indicates that a change in composition may have a similar disrupting effect on the crystal as a crystallographic twin. To study this effect further, a sharper compositional change was performed by rapidly altering the growth conditions to be more In rich (see section SI 1 in the Supporting Information for further details). This produced a multilayer stack in the nanowire shown in Figure 6. Figure 6a–c shows three frames extracted from the video where the 13-layers-thick multilayer segment grows to completion. Figure 6d–f shows the same process after GPA transformation of the frames. The GPA treatment color-codes the images based on the interplanar distance in the axial direction of the nanowire, and the result shows that the multilayer segment has a smaller interplanar distance than the rest of the nanowire displayed in the frames. GaAs has a smaller lattice than InAs for both crystal structures in which these nanowire materials grow (zincblende and wurtzite), which implies that the multilayer segment is Ga rich.^21^ Postgrowth XEDS was performed (switching to scanning transmission electron microscopy (STEM) mode) and is shown in Figure 6g, in the form of qualitative compositional maps. Here we can see the expected homogeneity of As in the nanowire as well as the localization of Au in the nanoparticle but more importantly that Ga shows a clear increase for a segment in the nanowire while In drops in the same region. This further confirms the formation of a heterostructure, and the location corresponds to the region grown in the multilayer growth mode. Interestingly, the change that initiated the multilayer growth was an increase in the In precursor flow, leading to the growth of a Ga-rich segment. One explanation could be that the increased In flux alters the compositional balance in the nanoparticle, forcing Ga out into the solid phase. As can be seen in the XEDS maps, after the multilayer segment, the nanowire again assumes an In-rich composition (just below the droplet).

### Multilayers in Conjunction with Kinking

**Figure 7:**
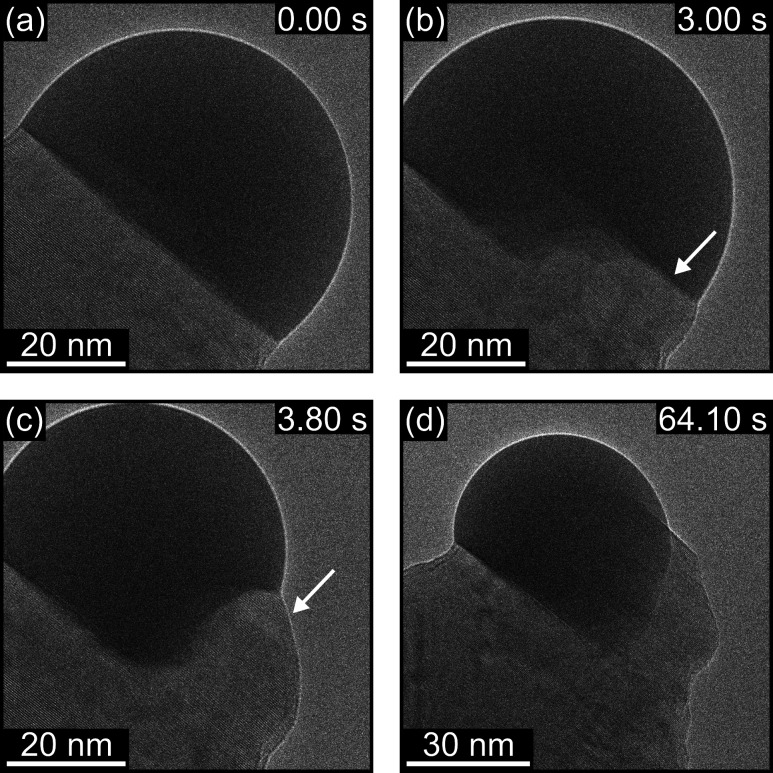
Initiation of a multilayer stack that leads to the displacement of the nanoparticle. (a) shows the nanowire before the multilayer nucleates. In (b) the nanoparticle still wets the flat interface at the top of the multilayer stack, while in (c) the stack has re-formed to remove the flat top facet. (d) shows a more demagnified micrograph after the nanoparticle has been further displaced. The time stamps in all frames indicate the time of the respective frames relative to panel (a).

A common problem in growing nanowires is that unfavorable growth conditions can lead to a change of growth direction, termed kinking. The presence of several incomplete layers at the growth front will affect the flatness of the interface between the nanoparticle and the nanowire, potentially displacing the droplet and changing the wetting angle. Therefore, it is important to investigate whether or not multilayer growth can have an effect on kinking behavior. Figure 7 shows an example of kinking, where the nanowire transitions from growing straight with a singular flat interface to wetting different facets and growing in another direction. Figure 7a shows the nanowire before the kinking event, while Figure 7b shows the situation when a multilayer segment has formed at the interface. The formation of the multilayer segment shown in Figure 7b is very rapid, and the individual nucleation events of the layers were not resolvable with our temporal resolution of 0.05 s. In this case, the multilayer growth is a transition state, before transitioning to the situation shown in Figure 7c. Whereas multilayer stacks typically have a top facet for the nanoparticle to wet (indicated by the arrow in Figure 7b), which has the same orientation as the original top facet of the nanowire, the multilayer segment has re-formed in Figure 7c to create a shape without the same clear top facet as the rest of the interface (indicated by the arrow in Figure 7c). Now the nanoparticle wets the side facet of the former multilayer stack, and this is where most of the growth will occur as the nanoparticle is displaced (Figure 7d)).

## Discussion

With the examples outlined in this paper, we have shown that multilayer growth is a phenomenon that occurs during growth of ternary InGaAs semiconductor nanowires. The individual observations range in magnitude from only 2 layers growing simultaneously up to multilayer stacks of more than 10 layers. What also becomes evident is that multilayer growth occurs as a response to some sort of perturbation, since the nanowire always grows in the regular layer-by-layer mode before and after the multilayer segment (unless it kinks). For the binary GaAs material system, Tornberg et al. have shown that the formation of crystallographic twins occasionally results in multilayer growth.^5^ There, the authors compared the change in total Gibbs free energy of the system during the growth of regular and twinned layers. Their conclusions showed that the layer propagation time of twinned layers is longer than for regular layers, which could lead to a supersaturation buildup between the nanoparticle and nanowire, since material is consumed at a lower rate. This in turn was speculated to be the reason for the occurrences of multilayers in conjunction with twins, since a higher supersaturation gives an increased nucleation probability. In the case of the ternary InGaAs nanowires described in this paper, multilayers have also been seen to emerge in conjunction with the formation of crystallographic twins. This leads us to believe that the origin is similar in the ternary case. However, multilayers seem to be more common, and extend to a larger number of layers, in the InGaAs case than for pure GaAs.

For a ternary nanowire another factor that can affect the growth is the composition in the nanowire. InAs and GaAs have a comparatively large lattice mismatch (about 7%),^22^ which is a reason it is difficult to form heterostructures of the pure binaries.^23^ A small compositional difference between solid layers could therefore cause a similar perturbation as a crystallographic twin, creating another route for multilayer initiation. This is likely the reason multilayers are more common for the ternary system analyzed here, compared to binary systems. For intermediate compositions there is also a miscibility gap in solid InGaAs at the temperatures typically used for nanowire growth.^10,24^ This means that these compositions are essentially metastable, which can lead to compositional fluctuations. The concentration of growth species in the nanoparticle is also important: the material flux into and out of the nanoparticle will determine the liquid composition, and fluctuations in the flow may affect the composition in the growing nanowire. Importantly, the concentration of Ga in the nanoparticle during the growth of InGaAs is small, meaning that it is more easily affected by fluctuations in the material flows. This can lead to drastic changes in the supersaturations of the two binaries throughout the growth, affecting their respective growth rate.^11^

One interesting aspect found in this study is the movement of material between the layers in a multilayer stack, described in Figure 5. One can imagine that, if compositional differences occur, the movement of material can act as a self-regulating effect, since the redistribution likely occurs as a response to lattice mismatch. Compositional fluctuations could then be smoothed out, in favor of a more uniform composition across layers. The extent of this effect is of course difficult to verify, since XEDS has neither the spatial nor the temporal resolution to measure the composition of layers as they grow and shrink *in situ*. However, if the self-regulating effect occurs, this can be an explanation for why InGaAs nanowires can grow in any composition within the miscibility gap of the system.^10^

We have also shown that multilayers can emerge in conjunction with the formation of a sharp heterostructure. This is of course a much larger perturbation to the system than a small change in composition or a crystallographic twin, so it is reasonable to assume that the effect is larger. As described in Figure 6, the multilayer segment that formed when the growth conditions were changed rapidly was larger than the other multilayer stacks discussed in this paper. Since the construction of heterostructures is one of the strengths of nanowires, and a common procedure in conventional growth, we expect that this situation is where the control of multilayers would be the most important. This is especially apparent for the GaAs to InAs transition, since there are few examples of successful heterostructure formation.^25,26^ Often, the heterostructure instead becomes less sharp with a ternary transition region, which again could be related to the redistribution of material discussed above. Examples of *ex situ* growth interruptions have shown the formation of islands at the interface between the nanoparticle and nanowire when GaAs to InAs heterostructures have been attempted.^16^ When the growth has been allowed to proceed further, droplet displacement and kinking has been the result. It seems plausible that the process in those studies was similar to what was presented in Figure 7, where the multilayer stack existed as a transition state before the nanowire kinked.

## Conclusions

In conclusion, we have demonstrated the presence of multilayer growth in the Au-seeded ternary In_*x*_Ga_1–*x*_As nanowire system. Compared to multilayers observed in binary systems, the size and frequency of multilayer stacks seems to be larger in the ternary system. Furthermore, the multilayers are highly dynamic, with the number of layers present in the stack changing rapidly during growth. Individual layers are also dynamic in the sense that material can be moved from already existing layers to rapidly complete a layer or nucleate a new one. This is expected to influence the composition of the final layers, while the overall growth rate is relatively unaffected. The initiation of multilayers seems to be related to perturbations in the crystal structure or composition. We have also shown the presence of multilayer nucleation in conjunction with kinking behavior in nanowires. We believe that in order to achieve full compositional control in growing ternary regions and heterostructures in nanowires, the multilayer growth phenomenon must be further considered and understood.

## Experimental Section

The nanowires were grown using metal–organic chemical vapor deposition (MOCVD) in an *in situ* setup, where the growth reactor is interfaced with an image-corrected (CEOS BCOR) Hitachi HF3300S transmission electron microscope, which allows the nanowires to be viewed during growth. The nanowires were grown from 30 nm diameter Au nanoparticles,^27^ deposited on micro electromechanical system (MEMS) heating chips made by Norcada Inc. These MEMS chips are equipped with a semi-electron-transparent SiN_*x*_ film exhibiting circular openings. Growth was initiated by heating the Au nanoparticles on the MEMS chips and supplying metal–organic and hydride precursors, containing the growth material. The precursors used in this study were trimethylindium (TMIn), trimethylgallium (TMGa), and arsine (AsH_3_), where H_2_ was used as a carrier gas for the metal–organics. Images and videos of the growth were recorded using a Gatan OneView IS camera, operated at 10–25 fps. For compositional analysis an Oxford Instruments SDD X-Max^N^ 80T XEDS system was used. Further details about the system are provided elsewhere.^28,29^

Pure GaAs growth was initiated at 420 °C by supplying TMGa and AsH_3_, producing a stem from where the growth could continue. The growth was continued until a sufficient length was achieved, i.e. until the tips of the nanowires had reached into the circular openings of the film. In the openings, the electron beam passes through unobstructed unless there is a nanowire blocking its path, thus enabling the best possible conditions for imaging. At this point, the temperature was lowered to 380 °C and TMIn supply was initiated, allowing the nanowire to transition from pure GaAs to a ternary In_*x*_Ga_1–*x*_As with varying composition. Details about the conditions used can be found in section SI 1 in the Supporting Information. The amount of In in the solid nanowire was thus allowed to increase throughout the growth of the nanowire. As the nanowires continued to grow, the composition of nanoparticles and nanowires, as well as the appearance of the growth interface, was monitored regularly via XEDS, imaging, and video acquisition.

## Supporting Information Available

Further experimental details and growth parameters, GPA analysis of the nanowire presented in Figure 3 in the main text, and an example of multilayer growth initiation without the presence of a crystallographic twin (PDF)
Section of Video 1 discussed in Figure 3 of the main text (AVI)
Section of Video 2 discussed in Figure 3 of the main text (AVI)
Section of Video 3 discussed in Figure 3 of the main text (AVI)

## Supplementary Material

ng2c00028_si_001.pdf

ng2c00028_si_002.avi

ng2c00028_si_003.avi

ng2c00028_si_004.avi
