# Supplementary material for: Observation of the Multilayer Growth Mode in Ternary InGaAs Nanowires
Source: ACS Nanosci Au. 2022 Aug 30;2(6):539–48. doi: 10.1021/acsnanoscienceau.2c00028 (PMC10125347; doi:10.1021/acsnanoscienceau.2c00028)
Supplement: Supplementary file 1 — ng2c00028_si_001.pdf [file ng2c00028_si_001.pdf]

Supporting Information for:
Observation of the multilayer growth mode in ternary InGaAs nanowires

Robin Sjökvist*[1,2], Marcus Tornberg[1,2], Mikelis Marnauza[1,2], Daniel Jacobsson[1,2,3], Kimberly A. Dick[1,2]

[1]Centre for Analysis and Synthesis, Lund University, Box 124, 22100, Lund, Sweden.

[2]NanoLund, Lund University, Box 118, 22100, Lund, Sweden.

[3]National Centre for High Resolution Electron Microscopy, Lund University, Box 124, 22100, Lund, Sweden.

* Email: robin.sjokvist@chem.lu.se

SI 1: Tabulated growth parameters

In Table SI-1, information about the growth parameters used in the experiments included in the main text and Supporting Information can be found. The table indicates which figures the experimental data have been included in, as well as the name of the accompanying Supporting Video, if applicable. The parameters include temperature, total pressure and partial pressures of the metal-organics and hydrides used in the study. The table also shows the calculated V/III supply ratio and In/III fraction.

For Figure 6, there are two sets of experimental parameters noted in Table SI-1. These detail the surrounding conditions before and after changing growth conditions, where the multilayer growth described in Figure 6 appeared within a minute after the change. Prior to the change in growth parameters, the environment was emptied of group III material in order to interrupt the growth. In total, the switch in experimental conditions occurred during a time span of a few minutes.

Table SI 1: Summary of experimental conditions used for the growth of the nanowires presented in the figures of the main text and Supporting Information. Supporting video name is supplied if applicable.

Used in Figure	Supporting video name	Temperature (°C)	Total pressure (Pa)	Partial pressure As (Pa)	Partial pressure Ga (µPa)	Partial pressure In (µPa)	V/III supply ratio	In/III fraction
3 (a)-(c), SI-1 (a), (d)	SI_Video1.avi	380	0.645	0.535	287	43	1621	0.130
3 (d)-(f), 5 (b)-(g), SI-1 (b), (e)	SI_Video2.avi	380	0.645	0.535	287	43	1621	0.130
3 (g)-(i), SI-1 (c), (f)	SI_Video3.avi	380	0.645	0.535	287	43	1621	0.130
6	-	380	1.000	0.830	308	68	2207	0.181
6	-	380	0.951	0.790	248	83	2386	0.251
1 (a)	-	380	0.226	0.187	69	15	2226	0.179
1 (b)	-	380	0.242	0.201	74	17	2209	0.187
SI-2	-	380	0.440	0.363	133	30	2227	0.184
7	-	380	1.096	0.910	445	12	1991	0.026

SI 2: GPA

Geometric phase analysis was performed on the nanowire shown in Figure 3 of the main text in order to investigate how the lattice spacing changes across the multilayer growth event. A frame was selected from each video (referred to as Video 1-3 in Figure 3 in the main text) to follow the growth of the nanowire and show how the lattice spacing evolves. The results are presented in Figure SI-1. As can be seen in the coloration of the geometric phase analysis in Figure SI-1 (d)-(f), the lattice spacing does not change dramatically throughout the multilayer growth. The main effect is that the appearance of the nanowire becomes more grainy as we lose contrast towards Figure SI-1 (f). The green arrows point to singular layers that have smaller lattice spacing, which is most likely an effect of stacking faults in the axial direction of the nanowire rather than compositional changes. Unless perfectly aligned to a zone axis, stacking faults can impose a slight shift in the lattice fringes used for the GPA, resulting in a different apparent lattice spacing.[1] The fact that singular layers appear to have smaller lattice spacing, and the nanowire is homogeneous in the surrounding regions, also suggests that the effect is caused by stacking faults rather than compositional changes.

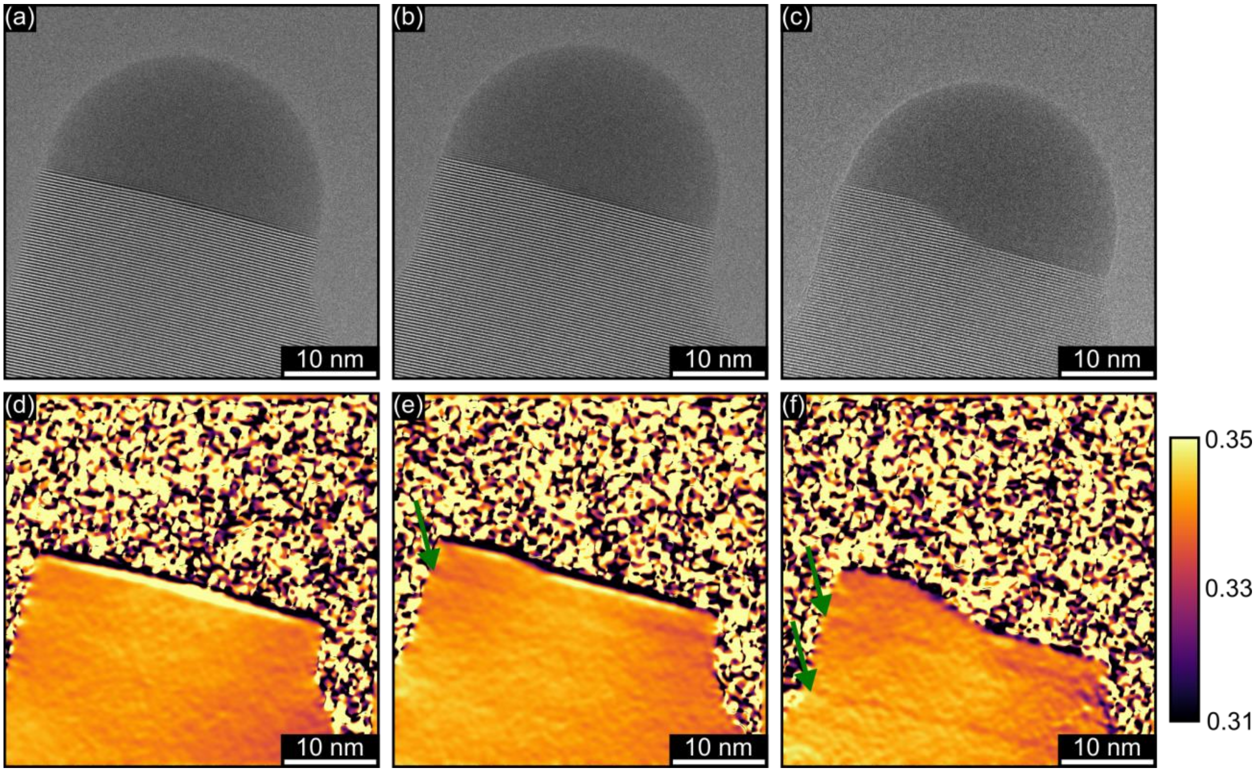

Figure SI-1: Frames extracted from the same videos as were used in Figure 3 of the main text, along with GPA representations of the same frames. (a)-(c) show the frames extracted from video 1, 2 and 3 respectively. (d)-(f) show the corresponding GPA treated frames. The color bar shows the lattice spacing in the axial direction of the nanowire. The coloration shows that the lattice spacing of the nanowire does not change much, except for the darker lines (marked with green arrows), attributed to stacking faults in the crystal.

SI 3: Multilayer without twin

Figure SI-2 shows a clear example of multilayer nucleation without the presence of a twin defect. This shows that twins are not a necessity for multilayer growth, and that other ways of initiation, e.g. compositional fluctuations, are possible explanations.

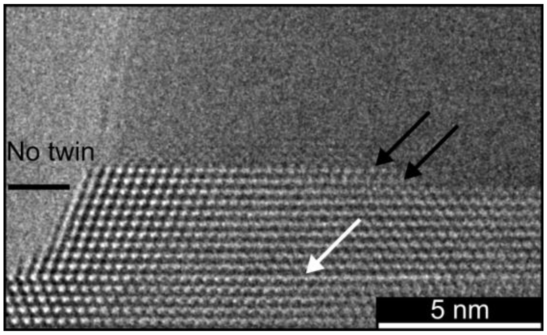

Figure SI-2: An example of a multilayer stack that has nucleated without the presence of a crystallographic twin. The black arrows point to the growth front of the two layers in the stack, while the dark line indicates the interface between the multilayer stack and the rest of the nanowire. An earlier crystallographic twin can be observed several layers down in the figure, indicated by the white arrow.

References

(1) Ek, M.; Leon, C.; Petersson, M.; Wallentin, J.; Wahlqvist, D.; Ahadi, A.; Borgström, M.; Wallenberg, R. Compositional Analysis of Oxide-Embedded III-V Nanostructures. *Nanotechnology* **2022**. https://doi.org/10.1088/1361-6528/AC75FA.
